# Genome-wide association analysis of wheat stem traits using 55K microarrays

**DOI:** 10.3389/fpls.2025.1635721

**Published:** 2025-07-24

**Authors:** Wei Wang, Na Sun, Kai Zhao, JiKun Song, Hui Fang, Guiqiang Fan, Yonghong Gao, Tianrong Huang, Yindeng Ding

**Affiliations:** ^1^ School of Computer Science and Information Engineering, Anyang Institute of Technology, Anyang, China; ^2^ Yili Prefecture Institute of Agricultural Science, Yining, China; ^3^ Cotton Research Institute, Chinese Academy of Agricultural Sciences, AnYang, China; ^4^ Institute of Crops, Xinjiang Academy of Agricultural Sciences, Xinjiang, China

**Keywords:** wheat, stem thickness, collapse, GWAS, candidate genes

## Abstract

Wheat lodging poses a severe threat to yield and quality, with the morphological and structural traits of the basal second internode being critical determinants of stem strength and lodging resistance. We conducted phenotypic analysis on 239 wheat varieties (lines) collected from around the world. This analysis was complemented by genotyping using the wheat 55K SNP chip. Genome-wide association analysis (GWAS) was executed employing the MLM (Q+K) algorithm within the TASSLE software suite. The findings unveiled pronounced phenotypic variability in the stem diameter of the second internode across disparate temporal intervals, characterized by a coefficient of variation spanning from 11.31% to 13.95%, alongside robust inter-year correlations. Furthermore, the genome-wide linkage disequilibrium (LD) decay distance was ascertained to 3 Mb. Analyses of population structure, phylogenetic dendrograms, and principal components revealed that the intrinsic population structure of the 239 wheat varieties (lines) was markedly simplified, segregating into three discernible subgroups. GWAS analysis identified 118 SNPs significantly associated with the stem diameter of the second internode *(P ≤ 0.001)*. Notably, among these loci, three SNPs (*AX-111557672*, *AX-94584919*, and *AX-109819835*) overlapped with previously reported associations, while the remaining 115 SNPs represented novel discoveries distributed across chromosomes 1A, 1B, 1D, 2A, 2B, 2D, 3A, 3B, 4A, 4B, 4D, 5A, 5B, 6A, 6B, 6D, 7A, 7B, and 7D. These newly identified loci exhibited substantial phenotypic variance explained, ranging from 8.09% to 29.14% for individual SNPs. Subsequent rigorous screening of loci showing significant phenotypic impacts and stability across diverse environmental contexts culminated in the identification of seven candidate genes implicated in the stem diameter of the second internode. This investigation provides new SNPs markers for enhancing lodging resistance in wheat, highlighting substantial practical implications.

## Introduction

In the context of escalating global food demands and climate change, ensuring the sustainable production of staple crops is critical for global food security. Wheat (*Triticum aestivum L.*), as one of the most widely cultivated cereal crops, accounts for approximately 21% of human dietary calories and 20% of protein intake (Wang et al., 2022a, b, 2024). Its unique gluten-forming proteins endow wheat-based products with distinct textures, making it indispensable in global diets (Liu et al., 2017, 2023). However, the pursuit of high-yield wheat breeding has led to a significant trade-off between yield potential and resistance to abiotic stresses. Lodging, defined as the displacement of the upright plant structure from its normal position, has emerged as a major constraint to wheat productivity. When wheat plants encounter adverse environmental conditions, such as strong winds during the grain-filling stage or waterlogging-induced soil instability, lodging can cause a yield reduction ranging from 10% to 40% (Zhu et al., 2008; Sun et al., 2017). Beyond yield losses, lodging compromises grain quality by reducing kernel plumpness, increases disease susceptibility, and complicates mechanized harvesting (Tian et al., 2017; Kong et al., 2024). Among different types of lodging, stem lodging, primarily characterized by the bending or breaking of basal internodes, is particularly prevalent during the milk-ripening stage (Anderson et al., 2025). Despite its economic significance, the genetic architecture underlying stem-related traits, such as internode strength, height, and mechanical properties, remains incompletely understood. Therefore, the study of genetic mechanisms of wheat stalk traits and improvement of stalk characteristics can help to improve the resistance of wheat to failure.

The second internode from the base of the stem is a critical support point for the wheat plant. Its morphological and structural characteristics have a significant impact on the plant’s lodging resistance (Gong, 1982; Kelbert et al., 2004; Zhang et al., 2016; Crook and Ennos, 1994). Research findings indicate that stem diameter, stem wall thickness, and mechanical strength at the second internode of the basal stem exhibit a significant positive correlation with lodging resistance (Rasheed et al., 2014). Plants with greater stem diameter and thicker stem walls generally exhibit enhanced lodging resistance. These features enhance the stem’s mechanical strength, allowing the plant to better endure external forces and resist bending or breaking under unfavorable conditions (Cat, 2024; Chen et al., 2024). Moreover, the composition of the cell wall at the second internode of the basal stem, particularly the content of cellulose and lignin, significantly influences lodging resistance. These structural components play a pivotal role in enhancing the mechanical properties of the stem, thereby improving its ability to withstand external stresses such as wind or heavy rainfall (Zhu et al., 2008; Tolossa et al., 2024; Xu et al., 2024). The higher the cellulose content of wheat stalks, the stronger the compressive strength of the stalks, while varieties with lower cellulose content are prone to collapse because their stalks are more fragile and weaker (Mostafaie et al., 2024). In recent years, the rapid development of molecular biology techniques has provided new perspectives for studying the genetic mechanisms of wheat resistance traits to failure (Niu et al., 2022). Researchers localized genetic loci associated with stalk traits through different populations and marker techniques (Khan et al., 2024). A total of 23 QTLs were identified on chromosomes 1B, 3B, 4A, 4B, and 5A, associated with traits such as stem strength, diameter, and pith cavity diameter. The phenotypic contribution of each QTL ranged from 3.5% to 44.0% (Dhariwal et al., 2022). Further research identified two QTLs associated with stem strength on chromosomes 3B and 5A. (Liu et al., 2017) using the 90K SNP chip, detected nine QTLs on chromosome 4B that regulate stem strength and the wall thickness at the second internode from the base, with phenotypic variation explained ranging from 9.4% to 36.6%. These findings elucidate the polygenic regulation of stem-related traits and identify chromosomal hotspot regions. In natural wheat populations, 37 SNP loci significantly associated with stem strength have been identified, distributed across 15 chromosomes (1A, 1B, 2B, 2D, 3A, 3B, 4A, 4B, 5A, 5B, 5D, 6B, 7A, 7B, and 7D). The phenotypic contribution rate of individual markers ranged from 7.76% to 13.77%, highlighting the complex genetic architecture underlying stem mechanical properties (Esposito et al., 2022). Analysis of 105 materials by 90K SNP microarrays identified multiple stem strength-associated loci on chromosomes 2B, 3B, 4A, 6B, and 7B, further confirming the synergistic multi-chromosome regulation (Genievskaya et al., 2020). The genes involved in the lignin biosynthesis pathway play a central role in the regulation of stem strength. Comparative transcriptomic analysis between the dwarf mutant DC20 and the wild-type (WT) wheat revealed 2135 differentially expressed genes in the stems during the booting stage. These genes primarily participate in sugar metabolism, energy metabolism, and post-transcriptional modification processes. Notably, 47 genes were significantly implicated in the dynamic balance of gibberellin (GAS) and auxin (IAA), cell elongation, and signal transduction (Bainsla et al., 2020). The study revealed that the expression levels of most differential genes were downregulated, while the expression of inhibitory factors was upregulated, suggesting that the dwarfism in the DC20 mutant may be closely associated with the suppression of hormone signaling and dysregulation of cell cycle control (Saint Pierre et al., 2010). Through these studies, scientists have continued to explore in depth the genetic mechanism of wheat resistance traits, providing a theoretical basis for further variety improvement and molecular breeding.

Although numerous studies have explored the genetic mechanisms underlying wheat lodging resistance, the genetic basis of morphological and structural characteristics in the second internode at the stem base remains systematically understudied. This study used 239 wheat varieties (lines) from around the world as research material to conduct a comprehensive analysis of morphological and structural traits, including stem diameter, at the second internode from the base. GWAS was conducted using the 55K SNP genotyping chip data for wheat, which covers 16,649 SNP markers across the entire genome. The analysis was performed using the mixed linear model (MLM, Q+K model) in the TASSEL software. Through GWAS, we aimed to identify significant SNP loci associated with stem diameter at the second internode from the base and further explore potential candidate genes. These findings will help to reveal the genetic basis of stem mechanical properties and provide targets for molecular breeding of lodging-resistant wheat varieties, and offer genetic resources for the development of lodging-resistant wheat varieties, thereby accelerating the breeding process for such varieties.

## Materials and methods

### Materials

This study selected 239 wheat varieties as experimental materials, covering multiple regions both domestically and internationally. Among them, 89.1% (213 varieties) were from China, primarily from Xinjiang (115 varieties), Beijing (34 varieties), Hebei (19 varieties), and Henan (19 varieties), with the remaining varieties distributed across Anhui, Jiangsu, Shandong, Shanxi, Shaanxi, and Tianjin provinces. The remaining 10.90% (26 varieties) were from abroad, with 25 varieties from the United States and 1 variety from Ukraine. All materials used in the study were able to adapt to the environmental conditions of the experimental site and completed their normal growth cycle.

### Experimental design

The study was conducted from September 2019 to July 2022 at two experimental sites of the Xinjiang Academy of Agricultural Sciences, namely the Zepu site (77°16’17.22”N, 38°11’21.65”E) and the Anningqu site (43°58’53.38”N, 87°30’17.72”E). Specifically, the Zepu site was designated as E1 in 2020, E2 in 2021, and E3 in 2022, while the Anningqu site was designated as E4 in 2020, E5 in 2021, and E6 in 2022. The six environmental conditions were tested over three years using a randomized block design, with two rows of each material planted per plot. The row length was 1 meter, the row spacing was 20 cm, and the sowing density was 350,000 seeds per acre. The planting direction was from north to south. Field management included control of light, water, fertilizer, and other routine practices. Each environmental condition was replicated three times to ensure consistency in cultivation and production conditions.

### Measurement of stem diameter at the second internode from the base

Following the milky stage of wheat, the first internode counted from the ground upward was designated as the basal first internode, and the second internode as the basal second internode. Using vernier calipers, five plants with comparable growth vigor from each experimental plot were measured, and their average value was calculated and recorded as the stem diameter of the basal second internode for the corresponding plot.

### Phenotyping methods

During the phenotypic analysis, variance analysis (ANOVA), descriptive statistics, significance tests for differences, and correlation analysis were conducted. All statistical procedures were performed using Python 3.8 in the Spyder IDE under Anaconda3, running on a Windows 10 workstation equipped with an Intel i7–6800 K 3.40 GHz CPU, 16 GB RAM, and a NVIDIA GeForce RTX 2080Ti GPU. The stem diameter traits of the basal second internode in wheat were analyzed using Pandas (v1.3.2), Matplotlib (v3.4.2), Scikit-learn (v0.24.2), and SPSS (v21.0) for statistical computation, correlation evaluation, and significance assessment.

### Association analysis

Association analysis, also termed association mapping or linkage disequilibrium (LD) mapping, is a quantitative genetic analytical technique. It integrates phenotypic diversity of target traits with polymorphisms in genes or markers, and identifies loci or markers associated with target traits based on LD between alleles at different loci in natural populations.

In this study, association analyses were conducted on wheat populations under diverse growing conditions using TASSEL v5.0 software, with integration of phenotypic evaluation datasets and a 55K SNP array. The Mixed Linear Model (MLM) was implemented for populations association profiling. Computational analysis revealed SNP markers exhibiting a significant association with phenotypic traits at a threshold of *P ≤ 0.001*. Subsequent visualization generated Manhattan plots and QQ plots. QQ plots validated analytical robustness by filtering spurious associations, while Manhattan plots mapped SNP distributions across wheat’s 21 chromosomes (horizontal axis) against −log10(P-value) (vertical axis), pinpointing loci with trait correlations.

### Candidate gene

The extended sequences of stable SNP markers were subjected to BLAST alignment in the Chinese Spring wheat genome database (https://urgi.versailles.inrae.fr/blast_iwgsc/). This was done to identify their locations and potential homologous sequences in the wheat genome. After obtaining the alignment results, gene function annotations were performed using the Wheat Omics 1.0 database (http://202.194.139.32/tools/intervalTools.html), which provided detailed information about the genes related to the SNP markers, including their functional roles, associated pathways, and other relevant biological information. This process helps to explore the genetic and functional significance of the identified SNP markers in wheat.

## Results

### Analysis of phenotypic variation in stem diameter at the second basal internode of wheat

**Figure 1:**
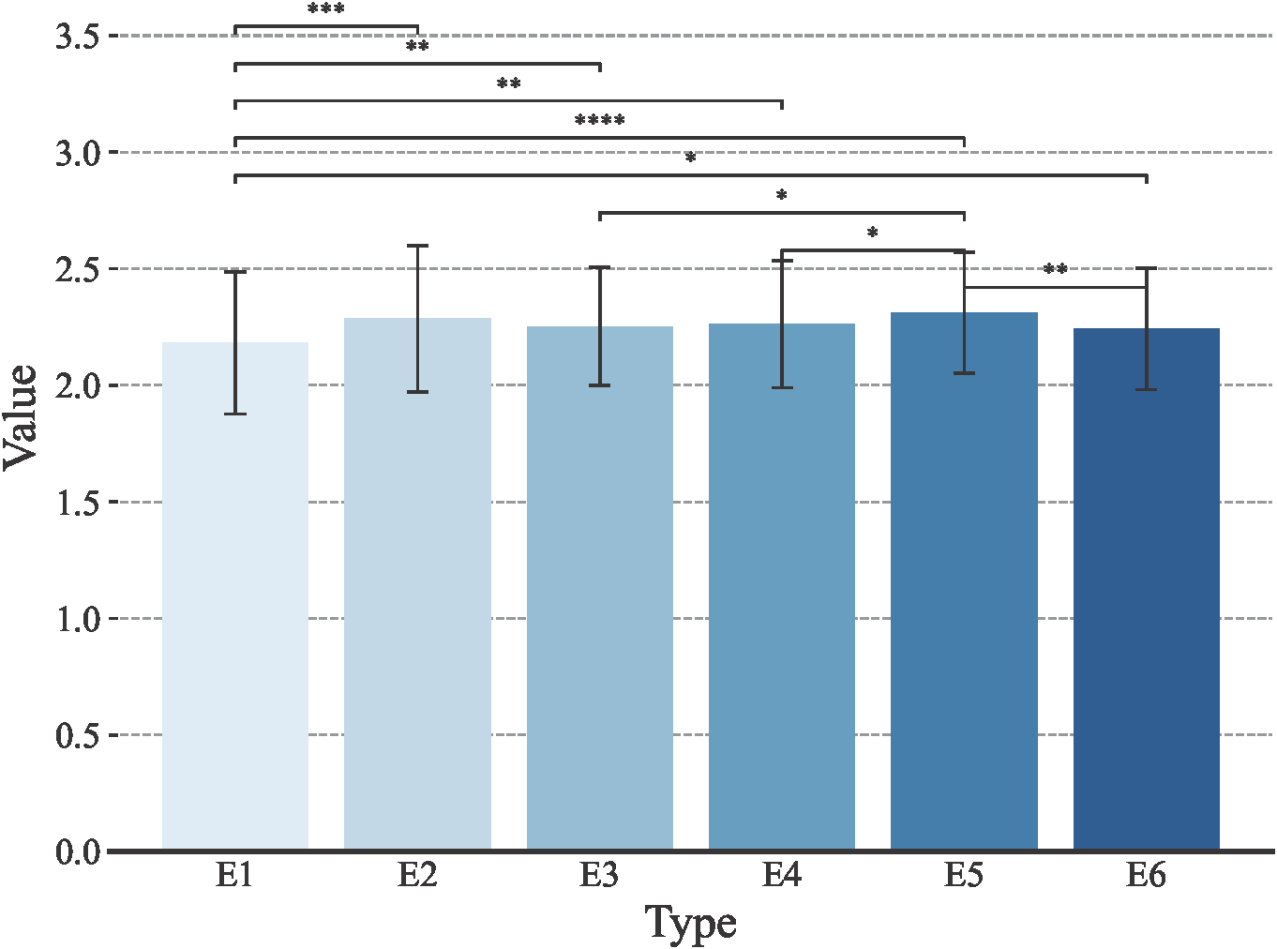
Trait distribution of the wheat stem diameter at the second internode from the base under different environmental conditions. E1: 2020 Zepu, Xinjiang; E2: 2021 Zepu, Xinjiang; E3: 2022 Zepu, Xinjiang; E4: 2020 Anningqu, Xinjiang; E5: 2021 Anningqu, Xinjiang; E6: 2022 Anningqu, Xinjiang. * p≤0.05; ** p≤0.01; *** p≤0.001; **** p≤0.0001.

Stem thickness of the 2nd internode at the base of wheat stalks was phenotyped at six environmental points, E1, E2, E3, E4, E5, and E6. The data were evaluated using four metrics: mean (μ), median, coefficient of variation (cv), and standard deviation (σ). As seen in **Figure 1**, the mean μ of stem thickness at the basal internode 2 of wheat stems in the E1 environment was 2.18, the median was 2.19, the coefficient of variation was 13.9%, and the standard deviation was 0.30; the mean μ of stem thickness at basal internode 2 of wheat stems in E2 environment was 2.29, median was 2.30, coefficient of variation was 13.7% and standard deviation was 0.31; the mean μ in the E3 environment was 2.25, the median was 2.27, the coefficient of variation was 11.3% and the standard deviation was 0.25; In the E4 environment, the mean (μ) was 2.26, with the median at 2.25, a coefficient of variation of 12.0%, and a standard deviation of 0.27. As for the E5 environment, it showed a mean (μ) of 2.31, a median of 2.33, a coefficient of variation of 11.2%, and a standard deviation of 0.26. The E6 environment, on the other hand, had a mean (μ) of 2.24, a median of 2.26, a coefficient of variation of 11.6%, and the standard deviation was 0.26. Overall, the data across different environments exhibit continuity and a normal distribution, which aligns with the typical characteristics of quantitative traits.

**Figure 2:**
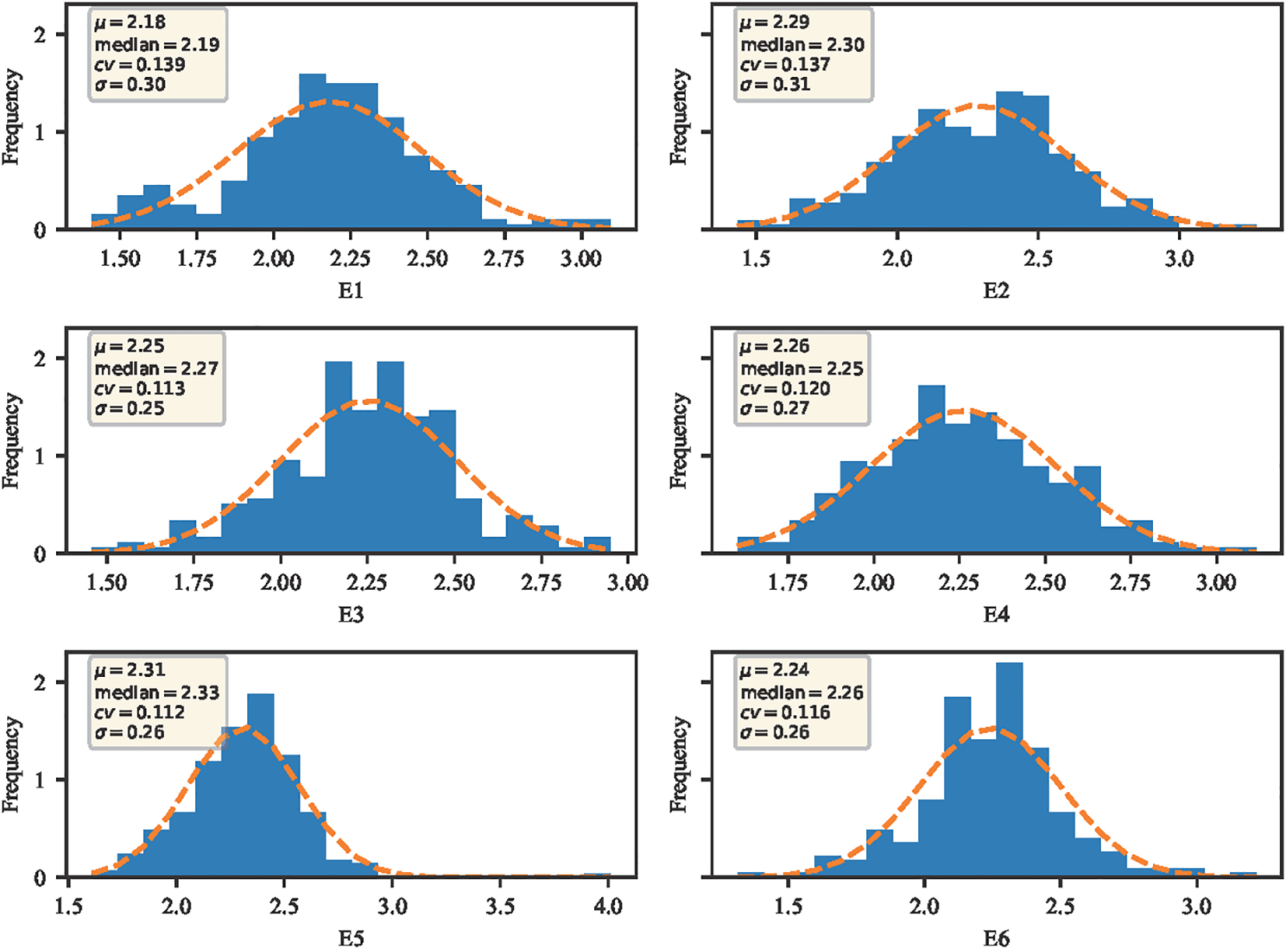
Statistical significance chart for the wheat stem diameter at the second internode from the base under different environmental conditions.

In the present investigation, we undertake a rigorous analysis that amalgamates significance testing, as delineated in **Figure 2**, with variance decomposition within a Best Linear Unbiased Prediction (BLUP) paradigm, as tabulated in **Table 1**. Our analytical framework has unveiled nuanced variability patterns in the second internode stem diameter of wheat culms. Empirical data demonstrate that this trait exhibits a standard deviation ranging from 0.16 to 0.31, with coefficients of variation fluctuating between 6.98% and 13.95%, and a heritability estimate of 0.73. A thorough variance analysis elucidates that genotypic disparities are the predominant contributors to the observed variation in stem diameter at the second internode, with environmental factors exerting a secondary influence. Collectively, these findings emphatically underscore the central role of genetic effects in orchestrating the phenotypic differentiation of this critical trait.

**Table 1:** Descriptive statistics for stem diameter trait at the second internode of wheat culm bases.

Envs	Min	Max	Mean	Standard deviation	Coefficient of variation	Heritability(h2)
	(cm)	(cm)	(cm)			
E1	1.41	3.09	2.18	0.3	13.95%	0.73
E2	1.43	3.27	2.29	0.31	13.73%	
E3	1.46	2.95	2.25	0.25	11.31%	
E4	1.6	3.12	2.26	0.27	12.03%	
E5	1.61	4.01	2.31	0.26	11.23%	
E6	1.31	3.22	2.24	0.26	11.61%	
Blup	2.23	3.13	2.29	0.16	6.98%	

### Correlation analysis of stem diameter traits at the second internode of wheat culm bases

**Figure 3:**
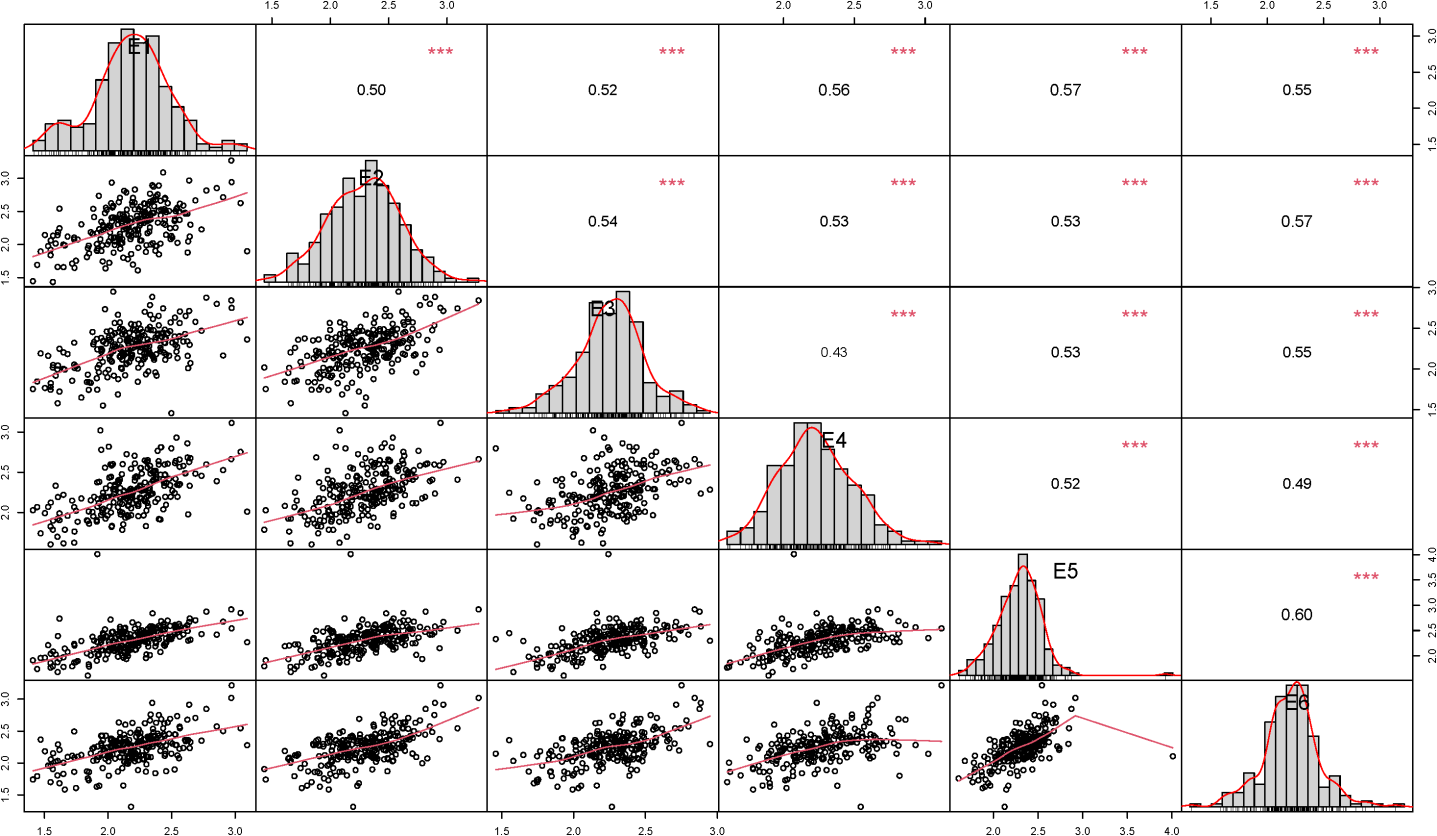
Correlation of stem thickness traits at the basal 2nd internode of wheat stalks in different environments.

In this study, the results of correlation analysis of stem thickness traits at the basal 2nd internode of wheat stalks at six experimental sites (E1-E6) are presented in **Figure 3**. The data showed that the correlation of this trait reached highly significant level *(p<0.001)* among all environmental points. The correlation coefficients for the stem thickness trait at the basal internode 2 of wheat stalks ranged from 0.50 to 0.57 in E1, 0.50 to 0.57 in E2, 0.43 to 0.54 in E3, 0.43 to 0.56 in E4, 0.52 to 0.60 in E5 and 0.49 to 0.60 in E6. Overall, the correlation coefficients of all environmental traits were distributed in the range of 0.43-0.60, indicating that this agronomic trait had stable and significant correlations under different environmental conditions.

### Population structure and evolutionary tree analysis

A comprehensive analysis of the population structure of 239 wheat varieties was performed using Structure software. Through a careful integration of population structure analysis, phylogenetic tree reconstruction and principal component analysis (PCA), we systematically partitioned the population structure into distinct subgroups. Our results, which were consistently supported by all three analytical approaches, indicate that it is both scientifically sound and methodologically appropriate to divide the total population into three subgroups: Subgroup 1, comprising 95 varieties (or lines); Subgroup 2, comprising 89 varieties (or lines); and Subgroup 3, comprising 55 varieties (or lines). The distribution frequencies of these subgroups are 39.75%, 37.24% and 23.01% respectively. In addition, we performed rigorous calculations to determine the linkage disequilibrium (LD) decay distances for the 239 wheat varieties (or lines) across genomes A, B, D and the entire genome, yielding values of 3 Mb, 3 Mb, 2 Mb and 3 Mb, respectively. Based on the LD decay distance observed for the whole genome, we have established a criterion whereby loci located within a 3 Mb interval on the physical map are designated as candidate loci for further analysis and investigation (Ding et al., 2023).

### GWAS analysis of stem thickness traits in the basal 2nd internode of wheat stalks

**Figure 4:**
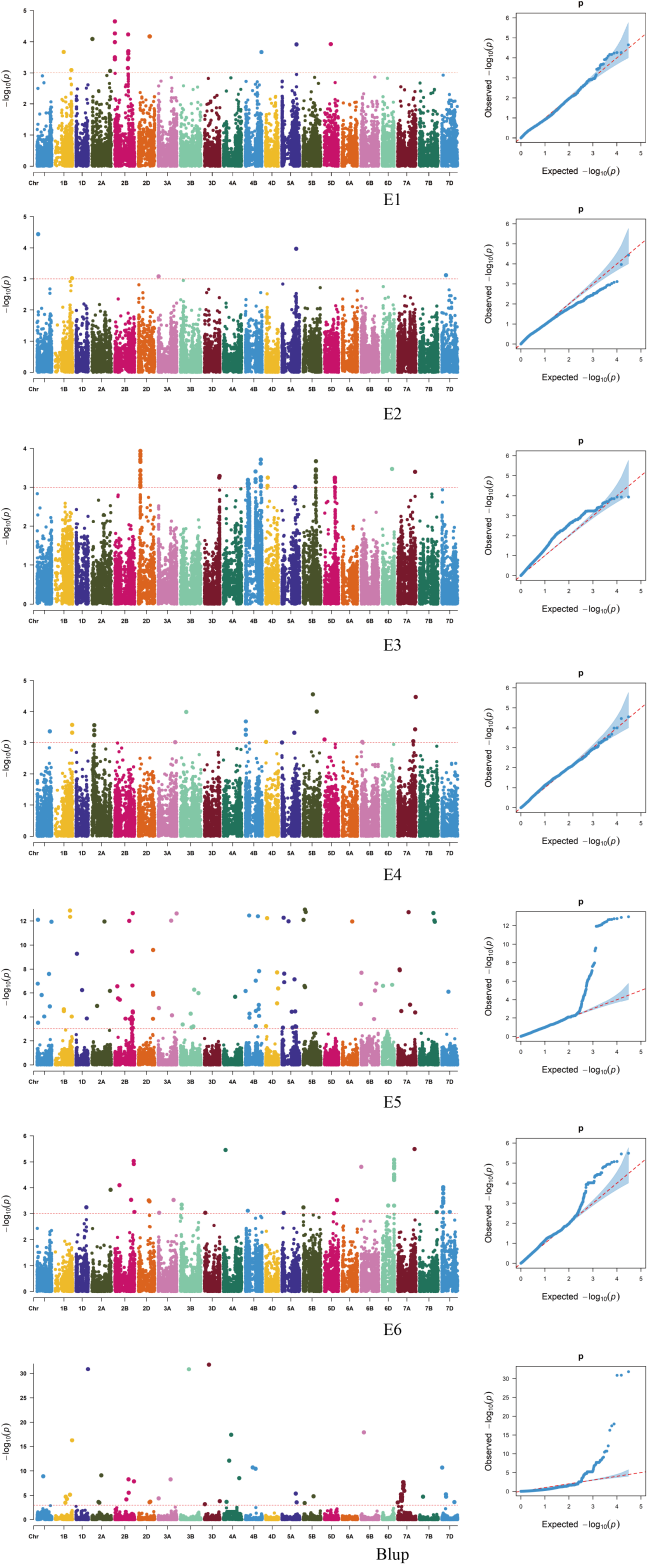
Manhattan plot of stem thickness traits in the basal internode 2 of wheat stalks in different environments.

A genome-wide association study (GWAS) was conducted using TASSEL 5.0 software, combining the stem thickness at the second internode from the base of 239 wheat varieties (lines) with 16,649 high-quality SNP markers identified from the 55K SNP array. Based on the MLM (Q+K) model, markers exhibiting a significance threshold of *P ≤ 0.001* were deemed significantly associated with the traits. Loci consistently detected across multiple environments were identified as stably inherited genetic regions (**Figure 4**; **Table 2**). GWAS Analysis of Wheat Stem Diameter at the Second Basal Internode. Based on genome-wide association study (GWAS) results, 139 single nucleotide polymorphism (SNP) markers were significantly associated with stem diameter at the second basal internode in wheat. These markers were distributed across 17 chromosomes: 1A, 1B, 1D, 2A, 2B, 2D, 3A, 3B, 4A, 4B, 4D, 5A, 5B, 6A, 6B, 6D, 7A, 7B, and 7D (**Figure 1**). Individual SNPs explained phenotypic variation rates ranging from 8.09% to 29.14%, highlighting their substantial contribution to trait heritability. *AX-111472018* and *AX-111589920*, located on chromosome 1B, are separated by only 0.2 Mb, with chromosomal clusters identified, and the phenotypic variation rate ranging from 10.42% to 10.75%. *AX-109449735*, localized on chromosome 2D, exhibits chromosome clustering and is positioned closer to other chromosomes within a region of reduced linkage disequilibrium (LD). The phenotypic variation rate for this marker ranges from 13.47% to 22.26%. *AX-11157475* and *AX-111490213*, located on chromosome 4D, are separated by 0.3 Mb, show chromosome clustering, and the phenotypic variation rate ranges from 11.85% to 17.81%. *AX-111502310* and *AX-110491224*, mapped to chromosome 5A, are separated by 0.8 Mb, with chromosomal clustering observed, and the phenotypic variation rates range from 15.90% to 17.57%. The remaining markers were detected in only one environment. Among the 118 identified SNPs, 115 novel loci were discovered including *AX-111089714* and *AX-109453515*, which have not been previously associated with stem thickness traits in the basal 2nd internode of wheat stalks.

**Table 2:** Information on significantly associated loci for stem thickness traits in the basal 2nd internode of wheat stalks.

Marker	Chr.	Position (Mb)	P-value	R2 (%)	Environment
*AX-94584919*	1A	39.20	7.9405E-13	28.49%	E5
*AX-111089714*	1A	579.82	1.1676E-12	28.07%	E5
*AX-109453515*	1A	482.33	2.5175E-08	17.51%	E5
*AX-109860252*	1A	30.82	1.6656E-07	14.11%	E5
*AX-110019998*	1A	186.66	1.4897E-06	12.01%	E5
*AX-111611028*	1A	513.89	0.000013189	9.97%	E5
*AX-109833703*	1A	47.59	0.00003656	10.23%	E2
*AX-110502416*	1A	289.33	0.000093299	9.38%	E5
*AX-109653547*	1B	587.61	1.2951E-13	28.52%	E5
*AX-109934481*	1B	592.85	4.4311E-13	29.14%	E5
*AX-111472018*	1B	342.66	0.000022825	10.75%	E5
*AX-111589920*	1B	342.97	0.00003191	10.42%	E5
*AX-110007940*	1B	659.99	0.000094673	9.37%	E5
*AX-111461657*	1D	38.02	5.2776E-10	21.50%	E5
*AX-111775865*	1D	243.41	5.8092E-07	14.35%	E5
*AX-111761576*	2A	505.42	1.1181E-12	28.12%	E5
*AX-109887603*	2A	724.74	6.8064E-07	14.20%	E5
*AX-108794432*	2A	209.24	0.000012126	11.36%	E5
*AX-110685697*	2A	23.55	0.000081812	8.23%	E1
*AX-89365114*	2B	716.82	2.152E-13	27.97%	E5
*AX-109347649*	2B	575.78	9.8414E-13	28.26%	E5
*AX-89589127*	2B	696.38	3.4176E-10	21.96%	E5
*AX-111577865*	2B	705.35	2.3646E-07	13.77%	E5
*AX-109051357*	2B	100.26	2.8032E-07	13.61%	E5
*AX-111488005*	2B	129.40	2.8499E-06	12.78%	E5
*AX-111057014*	2B	200.70	0.000003657	12.54%	E5
*AX-111071301*	2B	753.95	9.2288E-06	10.24%	E6
*AX-109816623*	2B	759.17	0.000011988	10.00%	E6
*AX-110615606*	2B	0.37	0.000022327	10.70%	E1
*AX-111080590*	2B	717.13	0.000034567	9.07%	E5
*AX-110023738*	2B	722.83	0.000042595	10.14%	E5
*AX-111478251*	2B	0.33	0.000054343	9.84%	E1
*AX-111500198*	2B	0.62	0.000054343	9.84%	E1
*AX-110527602*	2B	531.40	0.000058491	8.54%	E1
*AX-108830361*	2B	184.87	0.000080457	9.47%	E6
*AX-109827976*	2D	593.41	2.5774E-10	22.26%	E5
*AX-109449735*	2D	590.68	9.9158E-07	13.82%	E5
*AX-108867152*	2D	595.21	1.4153E-06	13.47%	E5
*AX-110637613*	2D	449.06	0.000067669	8.41%	E1
*AX-94417157*	2D	458.74	0.000067669	8.41%	E1
*AX-110906848*	3A	741.67	2.2704E-13	27.91%	E5
*AX-108938752*	3A	524.77	9.4013E-13	28.31%	E5
*AX-111074009*	3A	32.74	0.00001811	10.97%	E5
*AX-111024746*	3A	557.41	0.000072624	9.62%	E5
*AX-110379792*	3B	556.40	5.3703E-07	14.43%	E5
*AX-109889436*	3B	727.78	1.0481E-06	13.77%	E5
*AX-110047218*	3B	416.56	0.000053388	9.92%	E5
*AX-108972249*	4A	470.98	2.0856E-06	13.09%	E5
*AX-110990995*	4A	87.97	3.4954E-06	11.14%	E6
*AX-109932578*	4B	149.08	3.444E-13	29.42%	E5
*AX-109473487*	4B	500.83	3.9651E-13	29.26%	E5
*AX-111220208*	4B	548.38	1.4723E-08	18.06%	E5
*AX-111724067*	4B	413.50	9.1213E-08	16.21%	E5
*AX-111510337*	4B	6.94	6.9558E-07	14.18%	E5
*AX-110975969*	4B	535.34	0.00001002	10.22%	E5
*AX-111455852*	4B	527.41	0.000021974	10.78%	E5
*AX-111756841*	4B	418.65	0.000031309	10.44%	E5
*AX-110587188*	4B	163.36	0.000053229	9.92%	E5
*AX-111641369*	4B	13.90	0.000070898	9.59%	Blup
*AX-108737263*	4B	547.22	0.000083585	9.49%	E5
*AX-111719570*	4D	53.87	5.8065E-13	28.84%	E5
*AX-111490213*	4D	455.43	1.8719E-08	17.81%	E5
*AX-94404950*	4D	497.04	4.2731E-07	14.66%	E5
*AX-111157475*	4D	455.17	7.3334E-06	11.85%	E5
*AX-111541044*	5A	67.52	5.3883E-13	28.92%	E5
*AX-111513550*	5A	262.65	1.0649E-12	28.17%	E5
*AX-110491224*	5A	89.39	2.37E-08	17.57%	E5
*AX-111467228*	5A	503.40	7.0907E-08	16.46%	E5
*AX-111502310*	5A	90.21	1.238E-07	15.90%	E5
*AX-109438106*	5A	529.43	0.000034361	10.35%	E5
*AX-111221458*	5A	393.60	0.000036626	10.29%	E5
*AX-109381907*	5B	68.90	1.0764E-13	30.72%	E5
*AX-111504181*	5B	102.74	1.7124E-13	28.22%	E5
*AX-108749262*	5B	15.00	8.1877E-13	28.46%	E5
*AX-111583498*	5B	56.55	2.6139E-07	15.15%	E5
*AX-110933541*	5B	74.30	3.343E-07	14.91%	E5
*AX-110736785*	5B	389.13	0.000028113	10.48%	E4
*AX-89756375*	6A	409.80	1.1083E-12	28.13%	E5
*AX-111109562*	6B	22.24	1.9993E-08	17.75%	E5
*AX-110163419*	6B	618.26	1.6086E-07	15.64%	E5
*AX-111143764*	6B	565.08	6.3452E-07	14.27%	E5
*AX-111038900*	6B	4.57	8.4724E-06	10.38%	E5
*AX-109985395*	6B	14.75	0.000015515	11.05%	E6
*AX-109202589*	6D	392.03	2.0747E-07	15.38%	E5
*AX-94762593*	6D	25.64	2.5581E-07	15.17%	E5
*AX-108876425*	6D	470.57	8.2411E-06	10.34%	E6
*AX-111714196*	6D	470.38	8.4981E-06	11.64%	E6
*AX-110926979*	6D	470.74	0.000011304	10.05%	E6
*AX-110947659*	6D	470.94	0.000011304	10.05%	E6
*AX-110436530*	6D	471.27	0.000014253	11.14%	E6
*AX-111043108*	6D	470.75	0.000015009	11.09%	E6
*AX-111553486*	6D	470.34	0.000017578	10.93%	E6
*AX-109968823*	6D	469.21	0.000030347	10.41%	E6
*AX-110628600*	6D	469.39	0.000030347	10.41%	E6
*AX-109969797*	6D	470.58	0.000036899	10.22%	E6
*AX-109924298*	6D	470.60	0.000036899	10.22%	E6
*AX-110121576*	6D	470.88	0.000036899	10.22%	E6
*AX-110434749*	6D	470.94	0.000036899	10.22%	E6
*AX-110830333*	6D	470.94	0.000036899	10.22%	E6
*AX-109831577*	6D	470.95	0.000036899	10.22%	E6
*AX-110232069*	6D	469.56	0.000040704	10.12%	E6
*AX-110361087*	6D	470.39	0.000041283	10.11%	E6
*AX-111368581*	6D	470.39	0.000041283	10.11%	E6
*AX-108897553*	6D	470.39	0.000043682	10.06%	E6
*AX-109081485*	6D	470.35	0.000043755	10.05%	E6
*AX-110968183*	6D	470.75	0.000050757	9.91%	E6
*AX-111591978*	7A	436.18	1.7639E-13	28.19%	E5
*AX-109931165*	7A	75.21	1.0647E-08	18.39%	E5
*AX-111644940*	7A	83.15	1.243E-08	18.23%	E5
*AX-110360613*	7A	674.80	3.2214E-06	12.58%	E6
*AX-110505746*	7A	494.15	9.4888E-06	11.60%	E5
*AX-111109100*	7A	143.73	0.000032149	10.41%	E5
*AX-109644161*	7A	722.26	0.000034211	10.29%	E4
*AX-109320934*	7A	700.60	0.000041997	10.15%	E5
*AX-109106322*	7B	554.48	2.1031E-13	29.97%	E5
*AX-110668189*	7B	595.44	9.0943E-13	28.34%	E5
*AX-110050816*	7B	612.32	1.166E-12	28.07%	E5
*AX-111987065*	7D	267.01	7.9879E-07	12.60%	E5
*AX-109054953*	7D	56.87	0.000096137	8.09%	E6
*AX-110933671*	7D	56.93	0.000096137	8.09%	E6
*AX-110365192*	7D	56.98	0.000096137	8.09%	E6
*AX-108907765*	7D	57.40	0.000096137	8.09%	E6
*AX-111336287*	7D	57.45	0.000096137	8.09%	E6
*AX-109855959*	7D	57.47	0.000096137	8.09%	E6
*AX-110330229*	7D	57.67	0.000096137	8.09%	E6
*AX-110363071*	7D	57.72	0.000096137	8.09%	E6
*AX-86183329*	7D	57.78	0.000096137	8.09%	E6
*AX-110289031*	7D	57.79	0.000096137	8.09%	E6
*AX-109665227*	7D	57.81	0.000096137	8.09%	E6
*AX-109714466*	7D	57.83	0.000096137	8.09%	E6
*AX-111151622*	7D	57.84	0.000096137	8.09%	E6
*AX-109775040*	7D	57.87	0.000096137	8.09%	E6
*AX-110273752*	7D	57.87	0.000096137	8.09%	E6
*AX-109491313*	7D	58.05	0.000096137	8.09%	E6
*AX-109870585*	7D	58.16	0.000096137	8.09%	E6
*AX-111577495*	7D	58.19	0.000096137	8.09%	E6
*AX-111058442*	7D	58.20	0.000096137	8.09%	E6
*AX-111455688*	7D	58.26	0.000096137	8.09%	E6
*AX-109640417*	7D	58.33	0.000096137	8.09%	E6

### Functional prediction of candidate genes for stem thickness traits in the basal 2nd internode of wheat stalks

Significant SNP markers with substantial phenotypic effects and stable inheritance were retrieved from the Chinese Spring wheat genome database. Additionally, BLASTx sequence alignment was conducted against the NCBI database, leading to the identification of seven candidate genes most likely associated with the stem diameter at the second internode of the basal stem in wheat (**Table 3**). These candidate genes are primarily involved in the fibrillin family, calcium ion binding, disease resistance protein RPM1, and proteins containing the F-box domain. The gene *TraesCS1A02G422100*, located on chromosome 1A, encodes Asparagine synthase; *TraesCS1A01G058600*, also on chromosome 1A, is associated with Peroxisomal membrane 22 kDa (Mpv17/PMP22) family protein; *TraesCS2D01G488200* on chromosome 2D encodes Plastid-lipid associated protein PAP/fibrillin family protein; *TraesCS4D01G281500* on chromosome 4D encodes an F-box family protein; *TraesCS5A01G073300* on chromosome 5A encodes Disease resistance protein RPM1; *TraesCS6B02G050900* on chromosome 6B encodes a protein kinase catalytic domain; and *TraesCS7A02G481800* on chromosome 7A encodes a SNARE motif protein.

**Table 3:** Information of candidate genes for stem thickness trait in the 2nd internode at the base of wheat stalks.

Marker	Chr.	Position (Mb)	Genes	Gene annotation or encoded protein
*AX-111089714*	1A	579.819742	*TraesCS1A02G422100*	Asparagine synthase
*AX-94584919*	1A	39.199474	*TraesCS1A01G058600*	Peroxisomal membrane 22 kDa (Mpv17/PMP22) family protein
*AX-109449735*	2D	590.677285	*TraesCS2D01G488200*	Plastid-lipid associated protein PAP/fibrillin family protein
*AX-111157475*	4D	455.171184	*TraesCS4D01G281500*	F-box family protein
*AX-111502310*	5A	90.213921	*TraesCS5A01G073300*	Disease resistance protein RPM1
*AX-111109562*	6B	22.244496	*TraesCS6B02G050900*	Protein kinases, catalytic domain
*AX-110360613*	7A	674.802026	*TraesCS7A02G481800*	SNARE motif

## Discussion

### Association analysis of stem thickness traits in the basal second internode of wheat stalks

With the rapid development of biology and bioinformatics, GWAS (genome-wide association analysis) has become an important way to study quantitative traits in plants, and the mining of genes related to stem thickness traits in the 2nd internode at the base of wheat stalks has also been promoted to a greater extent (Li et al., 2024). Classical genetic studies have shown that stem diameter at the second internode of the basal stem in wheat is a complex trait controlled by both Mendelian and quantitative genes. This trait is closely associated with wheat lodging resistance, as the second internode at the base of the plant serves as a critical mechanical support, and its stem diameter directly affects the mechanical strength and bending resistance of the stem (Zhang et al., 2016; Song et al., 2021). This study conducted multi-environment genetic loci discovery across six environmental locations, identifying a total of 118 SNP markers distributed on chromosomes 1A, 1B, 1D, 2A, 2B, 2D, 3A, 3B, 4A, 4B, 4D, 5A, 5B, 6A, 6B, 6D, 7A, 7B, and 7D. The phenotypic variation explained by individual markers ranged from 8.99% to 85.48%. The widespread distribution of the 118 SNP markers suggests that the stem diameter trait in wheat is regulated by multiple genes, which may interact through a complex network to collectively influence the phenotype. The *AX-110671322* SNP marker located on chromosome 1D may be linked to the known lodging resistance gene Rht-B1b, while other markers may regulate cell wall biosynthesis or hormone signaling pathways, further affecting stem diameter development. In the 1960s, the application of dwarfing genes such as Rht1 and Rht2 significantly enhanced wheat lodging resistance and increased yield, triggering the agricultural ‘Green Revolution.’ Current research has confirmed that the Rht-B1 and Rht-D1 genes located on chromosomes 4B and 4D have become core genetic resources for global wheat breeding (Xu et al., 2023). Genome-wide studies have revealed that plant height-associated loci are widely distributed across all chromosomes except for 3D, 4A, 5D, 7A, 7B, and 7D. Notably, the *AX-109852602* locus on chromosome 7A overlaps with the *AX-111557672* locus reported by Yu et al. (2024), while the *AX-94584919* locus on chromosome 1A shows spatial overlap with the *AX-109819835* locus identified by Wang (2022). These overlapping regions suggest that they may have conserved functions in stem diameter regulation, providing new insights for the analysis of lodging resistance genetic mechanisms and the development of molecular markers.

### Functional analysis of candidate genes

Through the retrieval of stable genetic SNP markers in the Chinese Spring wheat genome database, seven candidate genes most likely associated with the second internode stem diameter at the base of the wheat stem were identified. These candidate genes are involved in several key biological processes, primarily related to the fibrillin family, calcium ion binding, the disease resistance protein RPM1, and F-box domain-containing proteins. The fibrillin family genes play a crucial role in cell wall construction and strengthening, while calcium ions are essential in cell signaling, regulating plant responses to environmental stresses. RPM1, as a resistance gene, is involved in wheat’s immune response to pathogens, and F-box proteins are important in plant protein degradation and signal transduction (Zhu et al., 2008; Xiong et al., 2021). The gene *TraesCS1A02G422100*, located on chromosome 1A, encodes Asparagine synthase, potentially regulating cytokinin precursor synthesis through amino acid metabolism, thereby affecting cell division and expansion. Another gene on chromosome 1A, *TraesCS1A01G058600*, is associated with Peroxisomal membrane 22 kDa (Mpv17/PMP22) family proteins. On chromosome 2D, *TraesCS2D01G488200* encodes a Plastid-lipid associated protein PAP/fibrillin family protein, which plays an important role in cell wall construction and reinforcement. Fibrillin proteins affect the mechanical strength of the stem cell wall through cellulose microfibril alignment and deposition. This finding complements previous results from associative transcriptomics, which suggested that xylan acetylation regulates stem strength by influencing the interaction of cell wall polysaccharides, while fibrillin family proteins mediate the structural composition of the cell wall (Miller et al., 2016; Zhou et al., 2023). This synergistic interaction indicates that the mechanical properties of the cell wall are a complex trait regulated by a multi-gene network, involving polysaccharide modifications and structural protein assembly. *TraesCS4D01G281500*, located on chromosome 4D, encodes an F-box family protein. This protein, a key component of the ubiquitin-proteasome degradation pathway, targets auxin signaling inhibitors (such as AUX/IAA proteins), regulating the expression of auxin-responsive genes (Harper et al., 2016; Miller et al., 2016). This finding complements previous studies on the involvement of auxin response factors (ARF) and the SAUR gene cluster in plant height regulation, suggesting that stem diameter and plant height may share overlapping hormonal signaling pathways. Additionally, the identification of calcium ion-binding genes further supports the role of calcium signaling in responding to environmental stimuli, such as mechanical stress, and regulating cell expansion, offering insights into the adaptive mechanisms of wheat stems under lodging stress. On chromosome 5A, *TraesCS5A01G073300* encodes the disease resistance protein RPM1. Its discovery suggests a potential link between disease resistance and stem development. RPM1 might indirectly modulate lignin deposition in the cell wall via the activation of the salicylic acid (SA) signaling pathway. It is well - documented that this pathway can induce the expression of genes implicated in lignin synthesis (Peng et al., 2014). This cross-regulation mechanism implies that wheat may enhance stem mechanical strength in response to pathogen infections by remodeling its stem structure, forming a synergistic defense and developmental strategy. Additionally, *TraesCS6B02G050900* on chromosome 6B encodes a protein kinase catalytic domain, likely a downstream component of the RPM1 signaling pathway, which may regulate cell wall-synthesizing enzymes via phosphorylation. Notably, TraesCS7A02G481800 on chromosome 7A encodes a protein containing the SNARE motif, which is involved in vesicle transport and may mediate the transport of cell wall precursors to the plasma membrane, providing new insights into improving stem diameter by regulating material transport efficiency.

The identified genes are involved in cell wall synthesis, cellulose accumulation, and plant hormone regulation. Genes related to fibrillin, carbohydrate synthesis, and cell division and expansion play a crucial role in stem diameter development (Kelbert et al., 2004; Peng et al., 2014). Moreover, the regulation of stem diameter is closely related to plant hormone balance, particularly the regulation of gibberellin and cytokinin. These findings provide more effective genetic improvement strategies for enhancing crop resistance to lodging, increasing yield, and improving stem physiological traits.

## Conclusion

In this study, a Q+K mixed linear model combined with a 55K SNP array was used to perform a genome-wide association analysis of stem diameter traits at the second internode of the basal stem in 239 wheat varieties (lines) from both domestic and international sources. A total of 118 SNP markers loci significantly associated with the trait (*P ≤ 0.001*) were successfully identified. Except for *AX-111557672*, *AX-94584919*, and *AX-109819835*, the other identified loci were not found in the previously reported articles and may be newly SNPs marker. These SNPs markers were distributed across multiple chromosomes of wheat, including 1A, 1B, 1D, 2A, 2B, 2D, 3A, 3B, 4A, 4B, 4D, 5A, 5B, 6A, 6B, 6D, 7A, 7B, and 7D. The phenotypic variance explained by individual SNP markers ranged from 8.09% to 29.14%. Furthermore, chromosomal clusters with large phenotypic effects were identified on chromosomes 2D, 4D, and 5A. By searching for stable, genetically significant SNP markers with large phenotypic effects in the Chinese Spring wheat genome database, seven candidate genes most likely related to the stem diameter trait at the second internode of the basal stem were ultimately selected. These findings provide essential theoretical support for further understanding the genetic mechanisms underlying stem diameter traits in wheat and offer potential molecular markers and target genes for wheat breeding improvements.

## Data Availability

The datasets presented in this study can be found in online repositories. The names of the repository/repositories and accession number(s) can be found in the article/supplementary material.
